# Association of selected adipokines with vitamin D deficiency in children with inflammatory bowel disease

**DOI:** 10.1186/s12887-024-04890-0

**Published:** 2024-07-03

**Authors:** Milos Geryk, Veronika Kucerova, Maria Velganova-Veghova, Hana Foltenova, Katerina Bouchalova, David Karasek, Martin Radvansky Jr., Eva Karaskova

**Affiliations:** 1 Department of Pediatrics, University Hospital Olomouc, Faculty of Medicine and Dentistry Palacky University Olomouc, Olomouc, Czech Republic; 2 Department of Clinical Biochemistry, University Hospital Olomouc, Olomouc, Czech Republic; 3 3rd Department of Internal Medicine - Nephrology, Rheumatology and Endocrinology, University Hospital Olomouc, Faculty of Medicine and Dentistry, Palacky University Olomouc, Olomouc, Czech Republic; 4 Department of Computer Science, Faculty of Electrical Engineering and Computer Science, VSB-Technical University of Ostrava, Ostrava - Poruba, Czech Republic

**Keywords:** Inflammatory bowel disease, Adiponectin, Resistin, Retinol-binding protein 4, Adipocyte fatty acid binding protein, Nesfatin-1, 25-hydroxyvitamin D

## Abstract

**Background:**

Adipose tissue is significantly involved in inflammatory bowel disease (IBD). Vitamin D can affect both adipogenesis and inflammation. The aim of this study was to compare the production of selected adipokines, potentially involved in the pathogenesis of IBD - adiponectin, resistin, retinol binding protein 4 (RBP-4), adipocyte fatty acid binding protein and nesfatin-1 in children with IBD according to the presence of 25-hydroxyvitamin D (25(OH)D) deficiency.

**Methods:**

The study was conducted as a case-control study in pediatric patients with IBD and healthy children of the same sex and age. In addition to adipokines and 25(OH)D, anthropometric parameters, markers of inflammation and disease activity were assessed in all participants.

**Results:**

Children with IBD had significantly higher resistin levels regardless of 25(OH)D levels. IBD patients with 25(OH)D deficiency only had significantly lower RBP-4 compared to healthy controls and also compared to IBD patients without 25(OH)D deficiency. No other significant differences in adipokines were found in children with IBD with or without 25(OH)D deficiency. 25(OH)D levels in IBD patients corelated with RBP-4 only, and did not correlate with other adipokines.

**Conclusions:**

Whether the lower RBP-4 levels in the 25(OH)D-deficient group of IBD patients directly reflect vitamin D deficiency remains uncertain. The production of other adipokines does not appear to be directly related to vitamin D deficiency.

## Background

Inflammatory bowel diseases (IBD) including Crohn’s disease (CD) and ulcerative colitis (UC) are considered to be more severe, more extensive and associated with more frequent complications in childhood [1, 2]. The etiopathogenesis of IBD is not well understood, but it results from an imbalance between genetic predisposition, environmental factors and alteration of the gut microbiome. It seems that visceral fat, especially mesenteric adipose tissue, is also involved in the pathogenesis of IBD, especially in patients with CD [3, 4].

Vitamin D is an important regulator of calcium and phosphorus metabolism related to bone health, but increasing evidence suggests its immunomodulatory abilities. It plays an important role in the composition of the intestinal microflora and also regulates immune homeostasis, especially the development and function of T cells and macrophages [5–7]. In addition, vitamin D is essential for maintaining the integrity of the mucosal barrier by protecting against infectious and inflammatory damage [8]. Suboptimal concentrations of vitamin D negatively affect the epithelial barrier and immune functions and lead to increased dysbiosis and possible translocation of intestinal bacteria, thereby influencing the onset and progression of IBD [9, 10]. Vitamin D deficiency is common in IBD patients [10, 11], but some studies also reported a lack of decrease in vitamin D levels in these patients [12, 13]. There are also different results in children with IBD pointing to an association between IBD severity, activity, therapeutic control and vitamin D levels [11–13].

Adipose tissue is an endocrine and immune organ that is significantly involved in inflammatory processes. Vitamin D affects adipogenesis, apoptosis, oxidative stress, and inflammation by suppressing the secretion of proinflammatory cytokines, including interleukin-6, tumor necrosis factor-α, and C-reactive protein, and by modulating the secretion of adipocytokines [14]. Adipokines are a group of mediators primarily released by adipocytes that modulate various metabolic functions in adipose tissue, liver, brain, muscle, pancreas, and the immune system. Regulatory immune function has been identified in many of them. The role of several adipokines in intestinal inflammation has recently been investigated [4, 15, 16]. The significance of vitamin D deficiency on abnormal production of adipokines in patients with IBD remains unclear.

The aim of this study was to compare the production of selected adipokines, potentially involved in the pathogenesis of IBD – adiponectin, resistin, retinol binding protein 4 (RBP-4), adipocyte fatty acid-binding protein (A-FABP), and nesfatin-1 [15–21] in affected children and healthy controls according to the presence of vitamin D deficiency.

## Methods

### Study design, inclusion and exclusion criteria

The study was undertaken as a case-control study of children with diagnosed IBD and healthy controls. The diagnosis of IBD was established for all patients according to the ESPGHAN Revised Porto Criteria for the diagnosis of IBD in children and adolescents [22]. Patients with ambiguous endoscopic and histological diagnoses were excluded. Any intercurrent illness, trauma, or other chronic inflammation were additional exclusion criteria. The control group consisted of sex- and age-matched healthy children. Height and weight were measured for all participants. Based on these parameters, the body mass index (BMI = weight (kg) / height (m)^2^) was calculated. According to age and sex, a Z-score for BMI was determined, which allows comparing this nutritional parameter between children of different age and gender. The Pediatric Crohn’s Disease Activity Index (PCDAI) in CD [23] and the Pediatric Ulcerative Colitis Activity Index (PUCAI) in UC patients [24] were used to determine IBD activity. Both PCDAI and PUCAI scores < 10 corresponded to IBD remission. A biomarker used to assess vitamin D status is the serum concentration of 25-hydroxyvitamin D (25(OH)D) as the sum of 25(OH)D2 and 25(OH)D3 [25]. According to the current classification, vitamin D deficiency (including pediatric patients) is defined as serum 25(OH)D values below 50 nmol/l [24, 26, 27].

### Laboratory analyses

Venous blood samples were drawn in the morning after a 12-h fast. Concentrations of adipokines were measured in the sample aliquots stored at − 80 °C, no longer than 6 months. To minimize the influence of pre-analytical factors on the concentration of calprotectin in stool, stool samples were kept in a refrigerator immediately after collection (i.e. at a temperature of 4–6 °C). The samples were delivered to the laboratory for analysis no later than the following day. Calprotectin concentrations were measured on the day the samples were delivered to the laboratory.

Blood counts were measured with an automated analyzer (Analyzer XN-3000™, Sysmex CZ s.r.o., Brno, Czech Republic). C-reactive protein (CRP) was assessed using an immunoturbidimetric method (kit Atellica CH CRP_2, Siemens Healthineers, Siemens Healthcare GmbH, Erlangen, Germany on an automatic analyzer Atellica Solutions, Siemens. Interleukin 6 was determined by immunochemiluminiscence method (kit Atellica IM IL6, Siemens Healthineers, Siemens Healthcare GmbH, Erlangen, Germany) on an automatic analyzer Atellica Solutions, Siemens. Ferritin was determined by chemiluminescence immunoassay (kit Atellica IM Ferritin, Siemens Healthineers, Siemens Healthcare GmbH, Erlangen, Germany) on an automatic analyzer Atellica Solutions, Siemens. Serum levels of 25-hydroxyvitamin D was detected by immunochemiluminiscence analysis (kit Atellica IM Vitamin D total, Siemens Healthineers, Siemens Healthcare GmbH, Erlangen, Germany) on an automatic analyzer Atellica Solutions, Siemens. The quantitative determination of serum calprotectin was performed using a lateral flow immunoassay (kit Quantum Blue MRP8/14, Bühlmann Laboratories AG, Schönenbuch, Switzerland) on a desktop analyzer Quantum Blue reader. Calprotectin in the stool was determined by an immunoturbidimetric method PETIA (kit BÜHLMANN fCal Turbo, Bühlmann Laboratories AG, Schönenbuch, Switzerland) on an automatic analyzer Atellica Solutions, Siemens. Adiponectin was determined by ELISA immunochemical kit: Human Adiponectin ELISA (Biovendor Laboratory Medicine Inc., Brno, Czech Republic), according to the manufacturer’s instructions. The antibodies used in this kit are specific for human adiponectin. Assay sensitivity was 26 ng/ml, precision CV was 4.9% (intra-assay) and 6.7% (inter-assay). Resistin was obtained by ELISA immunochemical kit Human Resistin ELISA (Biovendor Laboratory Medicine Inc., Brno, Czech Republic) according to the manufacturer’s instructions. The antibodies used in this ELISA are specific for human Resistin. Assay sensitivity was 0.012 ng/ml, precision CV was 5.9% (intra-assay) and 7.6% (inter-assay). The quantitative determination of RBP-4 was performed using ELISA immunochemical kit Human RBP4 (High Sensitivity) ELISA (Biovendor Laboratory Medicine Inc., Brno, Czech Republic) according to the manufacturer’s instructions. The antibodies used in this ELISA are specific for human RBP4. Assay sensitivity was 380 pg/ml, precision CV was 2.7% (intra-assay) and 5.0% (inter-assay). A-FABP was detected by immunoassay ELISA (kit Human FABP4 ELISA, Biovendor Laboratory Medicine Inc., Brno, Czech Republic), according to the manufacturer’s instructions. The antibodies used in this ELISA are specific for human A-FABP. Assay sensitivity was 0,08 ng/ml, precision CV was 2,5% (intra-assay) and 3,9% (inter-assay). Nesfatin-1 was measured by immunoassay ELISA (kit Human Nesfatin-1 ELISA, Biovendor Laboratory Medicine Inc., Brno, Czech Republic), according to the manufacturer’s instructions. The antibodies used in this ELISA are specific for human Nesfatin-1. Assay sensitivity was 0,021 ng/ml, precision CV was 4,25% (intra-assay) and 5,9% (inter-assay).

### Statistical analyses

Statistical analysis was performed using the R software (version 4.3.1). All values are expressed as medians and interquartile ranges (Q25 - Q75). Non-normal distribution was tested by Shapiro-Wilks test. Differences in variables between the groups were analyzed with the t-test and ANOVA for normally distributed variables, with the Mann–Whitney U-test and Kruskal-Wallis test for non-normally distributed variables. Fisher’s exact test was used to analyze categorical data. Spearman coefficient (ρ) was used to express the value of correlation. *P* < 0.05 was considered as statistically significant. Power analysis was performed using the “pwr” package in R, with a large effect size (0.8) and an alpha level set at 0.05. The analysis was performed and showed that our study has a power exceeding 87.7%, which corresponds to a high probability of detecting statistically significant differences between the groups compared.

## Results

### Basic characteristics of IBD patients and healthy controls

Seventy-four children with IBD [43 diagnosed with CD and 31 diagnosed with UC; age = 15 (13–15) years, number of boys = 42, number of girls = 32)] and 30 age-matched healthy controls [age = 15 (12–16), number of boys = 17, number of girls = 13)] met all inclusion criteria and avoided all exclusion criteria for this study. The majority of IBD patients were in remission (68.9% of IBD patients were in the inactive phase of the disease; 67.7% of UC patients had PUCAI < 10; 69.8% of CD patients had PCDAI < 10). Maintenance treatment in CD patients included: immunomodulators (azathioprine or methotrexate) – 35%, or biologics (infliximab or adalimumab) – 65%. Patients with UC were treated with mesalamine – 30%, immunomodulators (azathioprine) – 40%, or infliximab – 30%.

Table 1 shows the baseline clinical and laboratory characteristics of all IBD patients, individually with or without 25(OH)D deficiency, and healthy controls. There were no significant differences in BMI or Z scores for BMI between groups. Compared to healthy controls, IBD patients had significantly lower 25(OH)D levels. They had also higher levels of CRP, and amounts of fecal calprotectin (F-CPT) in the stool regardless of their 25(OH)D levels. Increased levels of IL-6 were detected only in IBD patients with 25(OH)D deficiency. No significant differences were found in investigated parameters (including CRP, IL-6 and F-CPT) between the IBD groups with and without 25(OH)D deficiency. No differences in vitamin D supplementation, and in history of Very Early Onset IBD (IBD presenting before 6 years of age) nor of Early Onset IBD (IBD presenting before 10 years of age) were detected between IBD patients with or without 25(OH)D deficiency – see Table 1. The following comorbidities were found in the group of children with IBD (with vitamin 25(0 H)D deficiency *versus* without vitamin 25(0 H)D deficiency): polyvalent allergy 22% (26% vs. 18%), bronchial asthma 7% (6% vs. 8%), atopic eczema 7% (6% vs. 8%), celiac disease 4% (6% vs. 3%), autoimmune liver disease 4% (6% vs. 3%), autoimmune thyroiditis 3% (3% vs. 3%), juvenile idiopathic arthritis 1% (0% vs. 3%). There were not significant differences in prevalence of recorded comorbidities between groups.

**Table 1:** Basic clinical and laboratory characteristics in IBD patients and healthy controls

	IBD group (*n* = 74)	IBD patients with 25(OH)D deficiency (*n* = 40)	IBD patients without 25(OH)D deficiency (*n* = 34)	healthy controls (*n* = 30)	*p*-value
Age (years)	15 (13–15)	15 (13–15)	15 (13–16)	15 (12–16)	n.s.
BMI (kg/m^2^)	20.3 (18.1–22.6)	19.6 (17.3–21.6)	20.4 (18.6–23.8)	19.6 (17.9–21.2)	n.s.
BMI Z-score	0.2 (-0.7-1.1)	0.02 (-0.9-0.9)	0.5 (-0.5-1.3)	0.1 (-0.6-0.8)	n.s.
CRP (mg/l)	1.1 (0.5–6.9)*	0.9 (0.5–8.1)^**c**^	1.2 (0.5–3.9)^**c**^	0.5 (0.4-3,9)^**a, b**^	**0.037**
IL-6 (ng/l)	2.4 (1.4–4.6)*	2.8 (1.4–4.6)^**c**^	1.7 (1.4–4.5)	1.4 (1.4–2.6)^**a**^	**0.003**
S-CPT (mg/l)	2.1 (1.5-3.0)	2.2 (1.6-3.0)	2.0 (1.5–2.7)	2.3 (1.4–3.6)	n.s.
F-CPT (µg/g)	389 (111–864)*	466 (146–1291)^**c**^	198 (99–546)^**c**^	49 (30–60)^**a, b**^	**< 0.001**
Ferritin (µg/l)	16.3 (8.0-36.5)	17.0 (8.1–33.5)	16.0 (9.3–37.5)	23.0 (15.1–32.5)	n.s.
thrombocytes (×10^9^/l)	318 (270–364)	317 (269–362)	319 (290–364)	283 (258–315)	n.s.
leucocytes (×10^9^/l)	7.2 (5.5-9.0)	7.1 (5.7–8.9)	7.3 (5.4–9.3)	7.1 (6.1–7.7)	n.s.
25(OH)D (nmol/l)	48.2 (36.1–64.3)*	36.6 (28.6–44.9)^**b, c**^	66.5 (57.5–73.0)^**a**^	58.3 (52.9–83.1)^**a**^	**< 0.001**
vitamin D supple- mentation (n)	35 (47%)	18 (53%)	17 (43%)	-	n.s.
VEO-IBD (n)	7 (9%)	5 (15%)	2 (5%)	-	n.s.
EO-IBD (n)	16 (22%)	9 (26%)	7 (18%)	-	n.s.

IBD = inflammatory bowel disease; BMI = body mass index (weight(kg)/height(m)^2^); CRP = C-reactive protein; IL-6 = interleukin-6; S-CPT = serum calprotectin; F-CPT = fecal calprotectin; 25(OH)D = 25-hydroxyvitamin D; VEO-IBD = Very Early Onset IBD (IBD presenting before 6 years of age); EO-IBD = Early Onset IBD (IBD presenting before 10 years of age)

Values are expressed as medians and interquartile ranges (Q25 - Q75). Difference between groups was tested by one-way ANOVA for normally distributed variables or Kruskal-Wallis test otherwise. Fisher’s exact test was used to analyze categorical data

In cases of significant difference pairwise tests were performed by t-test for normally distributed variables or to the Mann–Whitney U-test otherwise

*~ significant difference between IBD patients and healthy controls

^**a**^ ~ significant difference compared to IBD patients with 25(OH)D deficiency

^**b**^ ~ significant difference compared to IBD patients without 25(OH)D deficiency

^**c**^ ~ significant difference compared to healthy controls

### Selected adipokines in groups according vitamin D deficiency

Levels of resistin were significantly increased in IBD children compared to healthy controls regardless of 25(OH)D levels. IBD patients with 25(OH)D deficiency had significantly lower RBP-4 levels compared to healthy controls and also compared to IBD patients without 25(OH)D deficiency. Adiponectin levels were significantly reduced in children with IBD compared to healthy controls (especially in IBD patients without 25(OH)D deficiency), but no significant changes in adiponectin were found between IBD patients with and without 25(OH)D deficiency. There were also no significant differences in A-FABP4 or nesfatin-1 levels between any group - see Table 2. Table 3 shows correlations between resistin, RBP-4, adiponectin and other parameters in individual groups. In patients with IBD, resistin was positively correlated with markers of inflammation (CRP, IL-6, leukocytes, platelets), serum and fecal calprotectin. Adiponectin negatively correlated only with body mass index (BMI), Z-score for BMI, weight, and CRP. A significant correlation was also found between RBP-4 and BMI, weight, age, and 25(OH)D. Table 4 contains the values of adipokines in patients with active or inactive IBD, with or without 25(OH)D deficiency. Only a significant difference in RBP-4 was detected between active patients with and without 25(OH)D deficiency. Values of adipokines with or without 25(OH)D deficiency according to negative or positive Z-score for BMI in IBD patients are shown in Table 5. No statistically significant differences between groups were found.

**Table 2:** – Adipokines in individual groups

	All IBD patients (*n* = 74)	IBD patients with 25(OH)D deficiency (*n* = 40)	IBD patients without 25(OH)D deficiency (*n* = 34)	healthy controls (*n* = 23)	*p*-value
adiponectin (µg/ml)	10.8 (8.6–13.3)*	11.6 (9.1–13.4)	10.2 (8.5–12.4)^**c**^	12.5 (10.7–13.6)^**b**^	**0.027**
A-FABP (ng/ml)	14.4 (7.1–20.6)	13.9 (7.0–20.0)	14.6 (7.8–21.6)	15.1 (13.0-21.1)	n.s.
resistin (ng/ml)	6.3 (4.8–8.3)*	6.5 (5.0-8.6)^**c**^	5.7 (4.8–8.3)^**c**^	4.4 (3.3-6.0)^**a, b**^	**0.002**
RBP-4 (µg/ml)	35.0 (27.4–44.7)	32.8 (25.6–41.6)^**b, c**^	40.0 (29.2–56.4)^**a**^	40.9 (30.6–52.9)^**a**^	**0.047**
nesfatin-1 (pg/ml)	1135 (270–2305)	1040 (240–2320)	1310 (350–2270)	2065 (363–3663)	n.s.

IBD = inflammatory bowel disease; protein-1; A-FABP = adipocyte fatty acid-binding protein; RBP-4 = retinol-binding protein-4; 25(OH)D = 25-hydroxyvitamin D. Values are expressed as medians and interquartile ranges (Q25 - Q75). Difference between groups was tested by one-way ANOVA for normally distributed variables or Kruskal-Wallis test otherwise. In cases of significant difference pairwise tests were performed by t-test for normally distributed variables or to the Mann–Whitney U-test otherwise

*~ significant difference between IBD patients and healthy controls

^**a**^ ~ significant difference compared to IBD patients with 25(OH)D deficiency

^**b**^ ~ significant difference compared to IBD patients without 25(OH)D deficiency

^**c**^ ~ significant difference compared to healthy controls

**Table 3:** Correlations between baseline parameters and selected adipokines

		All IBD patients	IBD patients with 25(OH)D deficiency	IBD patients without 25(OH)D deficiency	healthy controls
**age**	**resistin**	0.07	-0.10	0.23	0.27
	**RBP-4**	**0.45**	0.23	**0.66**	**0.13**
	**adiponectin**	-0.09	0.03	-0.22	-0.05
**weight**	**resistin**	0.03	-0.08	0.09	0.16
	**RBP-4**	**0.24**	0.02	**0.39**	**0.78**
	**adiponectin**	**-0.25**	**-0.35**	-0.14	-0.23
**height**	**resistin**	0.11	0.01	0.13	0.23
	**RBP-4**	0.22	0.06	**0.35**	**0.71**
	**adiponectin**	-0.16	-0.24	-0.08	-0.15
**BMI**	**resistin**	-0.06	-0.24	0.11	0.10
	**RBP-4**	**0.23**	0.04	0.32	0.21
	**adiponectin**	**-0.26**	-0.28	-0.19	**-0.46**
**BMI** **Z-score**	**resistin**	-0.09	-0.21	0.06	-0.08
	**RBP-4**	0.1	-0.03	0.17	0.01
	**adiponectin**	**-0.27**	-0.3	-0.17	**-0.39**
**CRP**	**resistin**	**0.48**	**0.57**	**0.39**	0.27
	**RBP-4**	-0.05	-0.26	0.26	-0.35
	**adiponectin**	**-0.23**	-0.22	-0.30	-0.34
**IL-6**	**resistin**	**0.39**	**0.56**	0.25	0.32
	**RBP-4**	-0.06	-0.14	0.12	-0.33
	**adiponectin**	-0.07	-0.26	0.07	**-0.37**
**S-CPT**	**resistin**	**0.56**	**0.53**	**0.62**	0.29
	**RBP-4**	0.04	-0.15	0.27	0.15
	**adiponectin**	-0.21	-0.10	**-0.40**	**-0.40**
**F-CPT**	**resistin**	**0.40**	**0.40**	0.34	0.27
	**RBP-4**	-0.12	-0.15	-0.04	0.27
	**adiponectin**	-0.13	-0.15	-0.16	0.27
**ferritin**	**resistin**	0.09	0.13	0.08	0.01
	**RBP-4**	-0.15	0,00	-0.29	-0.01
	**adiponectin**	-0.14	**-0.36**	0.07	0.09
**thrombocytes**	**resistin**	**0.30**	**0.42**	0.23	0.01
	**RBP-4**	0.15	0,00	0.28	-0.14
	**adiponectin**	-0.13	-0.09	-0.19	0.16
**leucocytes**	**resistin**	**0.53**	**0.57**	**0.50**	0.34
	**RBP-4**	0.07	-0.20	0.32	-0.31
	**adiponectin**	-0.08	-0.17	0.02	-0.02
**25(OH)D**	**resistin**	-0.09	-0.14	0.03	-0.24
	**RBP-4**	**0.26**	0.21	-0.08	-0.19
	**adiponectin**	-0.13	0.12	-0.06	0.13

IBD = inflammatory bowel disease; BMI = body mass index; CRP = C-reactive protein; IL-6 = interleukin-6; S-CPT = serum calprotectin; F-CPT = fecal calprotectin; 25(OH)D = 25-hydroxyvitamin; RBP-4 = retinol-binding protein-4; values of Spearman coefficient (ρ) are highlighted in bold in case of statistical significance (*p* < 0.05)

**Table 4:** Adipokines in IBD patients with active or inactive disease

	adiponectin (µg/ml)	A-FABP (ng/ml)	resistin (ng/ml)	RBP-4 (µg/ml)	nesfatin-1 (pg/ml)
IBD patients with 25(OH)D deficiency and active disease (*n* = 12)	11.9 (8.95–12.38)	7.1 (4.9–17.3)	7.2 (5.5–9.9)	36.6* (31.1–40.4)	1425 (240–2863)
IBD patients without 25(OH)D deficiency and active disease (*n* = 11)	10.0 (5.8–12.0)	18.8 (6.2–20.9)	6.3 (5.2–12.6)	54.8 (38.4–64.6)	1480 (690–2305)
IBD patients with 25(OH)D deficiency and inactive disease (*n* = 28)	11.4 (9.1–13.5)	15.1 (10.4–21.0)	6.5 (4.85–7.9)	31.6 (25.2–41.6)	880 (240–2320)
IBD patients without 25(OH)D deficiency and inactive disease (*n* = 23)	10.5 (8.5–12.2)	13.8 (9.6–22.0)	5.7 (4.9–8.8)	37.7 (29.2–45.3)	875 (223–2213)

IBD = inflammatory bowel disease; protein-1; 25(OH)D = 25-hydroxyvitamin D; A-FABP = adipocyte fatty acid-binding protein; RBP-4 = retinol-binding protein-4

Values are expressed as medians and interquartile ranges (Q25 - Q75). Difference between groups was tested by Kruskal-Wallis test

*~ significant difference between 25(OH)D deficient and 25(OH)D non-deficient IBD patients separately in active or inactive disease groups

**Table 5:** Adipokines in IBD patients with negative or positive Z-score for BMI

	adiponectin (µg/ml)	A-FABP (ng/ml)	resistin (ng/ml)	RBP-4 (µg/ml)	nesfatin-1 (pg/ml)
IBD patients with 25(OH)D deficiency and negative Z-score for BMI (*n* = 20)	12.1 (10.7–15.3)	10.5 (6.4–17.3)	7.0 (5.3-9.0)	32.4 (27.8–39.9)	1315 (270–2050)
IBD patients without 25(OH)D deficiency and negative Z-score for BMI (*n* = 20)	11.6 (9.7–13.5)	13.0 (7.1–15.6)	5.70 (5.1–10.0)	36.4 (32.0-40.8)	1530 (365–2440)
IBD patients with 25(OH)D deficiency and positive Z-score for BMI (*n* = 11)	10.2 (7.7–12.8)	16.6 (12.8–20.9)	6.4 (4.4-8.0)	33.6 (25.2–41.8)	860 (164–3875)
IBD patients without 25(OH)D deficiency and positive Z-score for BMI (*n* = 23)	10.1 (6.7–11.7)	16.7 (9.6–22.6)	6.1 (5.0-8.1)	42.8 (29.2–57.2)	1255 (285–1845)

IBD = inflammatory bowel disease; protein-1; 25(OH)D = 25-hydroxyvitamin D; A-FABP = adipocyte fatty acid-binding protein; RBP-4 = retinol-binding protein-4

Values are expressed as medians and interquartile ranges (Q25 - Q75). Difference between groups was tested by Kruskal-Wallis test

*~ significant difference between 25(OH)D deficient and 25(OH)D non-deficient IBD patients separately in groups with negative or positive Z-score for BMI

## Discussion

IBD is commonly associated with vitamin D deficiency and altered adipokines production, but the results of this study did not support a direct link between these pathogenic mechanisms. Children with IBD had significantly higher resistin levels regardless of 25(OH)D levels. IBD patients with 25(OH)D deficiency only had significantly lower RBP-4 levels compared to healthy controls and also compared to IBD patients without 25(OH)D deficiency. However, no significant differences in other adipokines levels were found between pediatric IBD patients with or without 25(OH)D deficiency.

Many studies have found that circulating resistin levels are increased in IBD patients [16, 17, 19, 28, 29], including children [16, 30], and that resistin levels decrease in IBD patients during anti-inflammatory treatment [19]. Resistin, although described as an adipocyte-derived cytokine, is highly expressed in neutrophils and macrophages and is released upon stimulation by inflammatory stimuli [31]. This may indicate that it is a non-specific marker of inflammation. Resistin was positively correlated with inflammatory markers in this study. In addition, resistin was also correlated with serum and fecal calprotectin, which may indicate its specific role in intestinal involvement. Recently, resistin, along with elastase and lactoferrin, were identified as potential plasma biomarkers of pediatric IBD based on a comprehensive proteomic screen [30]. The results of the presented study did not show a significant association between resistin and 25(OH)D levels. A negative association between resistin and vitamin D levels was found in obese patients [32, 33], but not in all cases [34], including also obese children [35]. In patients with IBD, inflammation itself appears to be the main cause of elevated circulating resistin levels regardless of vitamin D levels or body mass index.

Lower RBP-4 levels in IBD patients with 25(OH)D deficiency probably reflected lower RBP-4 production. There were positive associations between RBP-4 and 25(OH)D levels in all IBD patients (especially in the group with 25(OH)D deficiency). RBP-4 is mainly expressed in the liver followed by robust expression in adipose tissue. Thus, RBP-4 should be considered primarily a hepatokine rather than an adipokine, and liver expression reflects retinoid stores in the body [36]. There are varying reports on RBP-4 levels in IBD patients. Elevated serum levels of RBP-4 have been found in adults with IBD [17]. Conversely, other studies found no difference in RBP-4 levels between pediatric IBD patients and healthy controls [16, 18]. In our study, a huge and significant difference in RBP-4 was documented especially in individuals with active IBD, and RBP-4 negatively correlated with activity of IBD, which could suggest a protective anti-inflammatory mechanism of action in addition to vitamin A transport [18]. A possible explanation for the decrease of RBP-4 in IBD patients with 25(OH)D deficiency in our study could be the coincidence of vitamin D and vitamin A deficiency as another fat-soluble vitamin. A positive association of RBP-4 with a reduced level of circulating 25-(OH)D was also found in obese children and adolescents [37]. Whether there is a direct effect of vitamin D on RBP-4 remains uncertain.

No significant differences in other adipokines were found in IBD children with or without 25(OH)D deficiency. Serum adiponectin levels were found to be decreased in all IBD patients. Interestingly, patients without 25(OH)D deficiency had the lowest adiponectin levels. Controversial reports of adiponectin levels - decreased [16, 17, 29, 38–40], increased [28] or unchanged [41–43] - have been reported in many studies with IBD individuals. The discrepancy in these results may be partly explained by the small cohorts used in some studies, the different treatment status of patients or disease activity. The results suggest that adiponectin plays an important role in maintaining intestinal homeostasis, but its precise effect on intestinal inflammation remains unclear [4, 15, 21]. Reports on the relationship between adiponectin and vitamin D deficiency are also controversial. Some authors found a significant association between vitamin D deficiency and adiponectin levels [44–48], some did not [49–51]. A recent meta-analysis showed that vitamin D intake does not have a statistically significant effect on serum adiponectin concentration [52]. In this study, 25(OH)D did not correlate with adiponectin. Additionally, no association was found between 25(OH)D and other adipokines (A-FABP, nesfatin-1) or markers of inflammation. Therefore, vitamin D deficiency does not appear to be directly related to adverse adipokine production in the dysfunctional adipose tissue of children with IBD.

This study has some limitations. Due to the cross-sectional design, the study could only demonstrate significant correlations, but not potential causality of the relationships found. In addition to the relatively small size of the patient cohorts (which is common for pediatric cohorts), a limitation is the fact that the majority of IBD patients were successfully treated and were in remission. Low disease activity was also accompanied by good nutritional status of IBD patients (they did not differ in BMI or Z-score for BMI from healthy controls). These facts may have influenced adipokines production as well as vitamin D levels. Further prospective studies will be needed to determine the effect of long-term monitoring of disease activity and different therapeutic approaches, including the effect of vitamin D administration.

## Conclusion

In summary, significantly lower levels of adiponectin and higher levels of resistin reflect the persistence of dysfunctional adipose tissue regardless of 25(OH)D levels. Whether the lower RBP-4 levels in the 25(OH)D-deficient group of IBD patients directly reflect vitamin D deficiency remains uncertain. The production of other adipokines does not appear to be directly related to vitamin D deficiency.

## Data Availability

Data is provided within the manuscript. Sequence data that support the findings of this study and are not openly available due to reasons of sensitivity and are available from the corresponding author upon reasonable request. Data are located in controlled access data storage at University Hospital Olomouc.

## References

[1] Bousvaros A (2010). Use of immunomodulators and biologic therapies in children with inflammatory bowel disease. Expert Rev Clin Immunol.

[2] Ruel J, Ruane D, Mehandru S, Gower-Rousseau C, Colombel JF (2014). IBD across the age spectrum—is it the same disease?. Nat Rev Gastroenterol Hepatol.

[3] Gonçalves P, Magro F, Martel F (2015). Metabolic inflammation in inflammatory bowel disease: crosstalk between adipose tissue and bowel. Inflamm Bowel Dis.

[4] Karaskova E, Velganova-Veghova M, Geryk M, Foltenova H, Kucerova V, Karasek D (2021). Role of adipose tissue in inflammatory bowel disease. Int J Mol Sci.

[5] Gubatan J, Moss AC (2018). Vitamin D in inflammatory bowel disease: more than just a supplement. Curr Opin Gastroenterol.

[6] Yoon JY (2019). Nutritional approach as therapeutic manipulation in inflammatory bowel disease. Intest Res.

[7] Rigterink T, Appleton L, Day AS (2019). Vitamin D therapy in children with inflammatory bowel disease: a systematic review. World J Clin Pediatr.

[8] Meeker S, Seamons A, Maggio-Price L, Paik J (2016). Protective links between vitamin D, inflammatory bowel disease and colon cancer. World J Gastroenterol.

[9] Leskovar D, Meštrović T, Barešić A, Kraljević I, Panek M, Čipčić Paljetak H (2018). The role of vitamin D in inflammatory bowel disease - assessing therapeutic and preventive potential of supplementation and food fortification. Food Technol Biotechnol.

[10] Del Pinto R, Pietropaoli D, Chandar AK, Ferri C, Cominelli F (2015). Association between inflammatory bowel disease and vitamin D deficiency: a systematic review and meta-analysis. Inflamm Bowel Dis.

[11] Fatahi S, Alyahyawi N, Albadawi N, Mardali F, Dara N, Sohouli MH (2023). The association between vitamin D status and inflammatory bowel disease among children and adolescents: a systematic review and meta-analysis. Front Nutr.

[12] Sun YH, Tian DD, Zhou JM, Ye Q (2023). Association between vitamin D level and pediatric inflammatory bowel disease: a systematic review and meta-analysis. Front Pediatr.

[13] Veit LE, Maranda L, Fong J, Nwosu BU (2014). The vitamin D status in inflammatory bowel disease. PLoS ONE.

[14] Szymczak-Pajor I, Miazek K, Selmi A, Balcerczyk A, Śliwińska A (2022). The Action of Vitamin D in adipose tissue: is there the link between vitamin D Deficiency and Adipose tissue-related metabolic disorders?. Int J Mol Sci.

[15] Weidinger C, Ziegler JF, Letizia M, Schmidt F, Siegmund B (2018). Adipokines and their role in intestinal inflammation. Front Immunol.

[16] Karaskova E, Kubickova V, Velganova-Veghova M, Geryk M, Foltenova H, Karasek D (2022). Circulating levels of WISP-1 (Wnt1-inducible signaling pathway protein 1) and other selected adipokines in children with inflammatory bowel disease. Physiol Res.

[17] Valentini L, Wirth EK, Schweizer U, Hengstermann S, Schaper L, Koernicke T (2009). Circulating adipokines and the protective effects of hyperinsulinemia in inflammatory bowel disease. Nutrition.

[18] Roma E, Krini M, Hantzi E, Sakka S, Panayiotou I, Margeli A, Papassotiriou I, Kanaka-Gantenbein C (2012). Retinol binding protein 4 in children with inflammatory bowel disease: a negative correlation with the disease activity. Hippokratia.

[19] Karmiris K, Koutroubakis IE, Xidakis C, Polychronaki M, Kouroumalis EA (2007). The effect of infliximab on circulating levels of leptin, adiponectin and resistin in patients with inflammatory bowel disease. Eur J Gastroenterol Hepatol.

[20] Beyaz Ş, Akbal E (2022). Increased serum nesfatin-1 levels in patients with inflammatory bowel diseases. Postgrad Med J.

[21] Morshedzadeh N, Rahimlou M, Asadzadeh Aghdaei H, Shahrokh S, Reza Zali M, Mirmiran P (2017). Association between adipokines levels with inflammatory bowel disease (IBD): systematic reviews. Dig Dis Sci.

[22] Levine A, Koletzko S, Turner D, Escher JC, Cucchiara S, de Ridder L (2014). European Society of Pediatric Gastroenterology, Hepatology, and Nutrition. ESPGHAN revised porto criteria for the diagnosis of inflammatory bowel disease in children and adolescents. J Pediatr Gastroenterol Nutr.

[23] Hyams J, Markowitz J, Otley A, Rosh J, Mack D, Bousvaros A, Pediatric Inflammatory Bowel Disease Collaborative Research Group (2005). Evaluation of the pediatric crohn disease activity index: a prospective multicenter experience. J Pediatr Gastroenterol Nutr.

[24] Sempos CT, Heijboer AC, Bikle DD, Bollerslev J, Bouillon R, Brannon PM et al. Vitamin D assays and the definition of hypovitaminosis D: results from the First International Conference on Controversies in Vitamin D. Br J Clin Pharmacol. 2018;84(10):2194–2207. 10.1111/bcp.13652 PMC6138489 29851137

[25] Turner D, Hyams J, Markowitz J, Lerer T, Mack DR, Evans J (2009). Pediatric IBD Collaborative Research Group. Appraisal of the pediatric ulcerative colitis activity index (PUCAI). Inflamm Bowel Dis.

[26] Holick MF (2007). Vitamin D deficiency. N Engl J Med.

[27] Misra M, Pacaud D, Petryk A, Collett-Solberg PF, Kappy M (2008). Drug and Therapeutics Committee of the Lawson Wilkins Pediatric Endocrine Society. Vitamin D deficiency in children and its management: review of current knowledge and recommendations. Pediatrics.

[28] Karmiris K, Koutroubakis IE, Xidakis C, Polychronaki M, Voudouri T, Kouroumalis EA (2006). Circulating levels of leptin, adiponectin, resistin, and ghrelin in inflammatory bowel disease. Inflamm Bowel Dis.

[29] Konrad A, Lehrke M, Schachinger V, Seibold F, Stark R, Ochsenkühn T (2007). Resistin is an inflammatory marker of inflammatory bowel disease in humans. Eur J Gastroenterol Hepatol.

[30] Louis Sam Titus ASC, Vanarsa K, Soomro S, Patel A, Prince J, Kugathasan S (2023). Resistin, Elastase, and Lactoferrin as potential plasma biomarkers of Pediatric Inflammatory Bowel Disease based on Comprehensive Proteomic screens. Mol Cell Proteom.

[31] Boström EA, Tarkowski A, Bokarewa M (2009). Resistin is stored in neutrophil granules being released upon challenge with inflammatory stimuli. Biochim Biophys Acta.

[32] Stokić E, Kupusinac A, Tomic-Naglic D, Smiljenic D, Kovacev-Zavisic B, Srdic-Galic B (2015). Vitamin D and dysfunctional adipose tissue in obesity. Angiology.

[33] Tariq S, Tariq S, Khaliq S, Baig M, Murad MA, Lone KP (2021). Association between Vitamin D and resistin in postmenopausal females with altered Bone Health. Front Endocrinol (Lausanne).

[34] Vilarrasa N, Vendrell J, Maravall J, Elío I, Solano E, San José P (2010). Is plasma 25(OH) D related to adipokines, inflammatory cytokines and insulin resistance in both a healthy and morbidly obese population?. Endocrine.

[35] Roth CL, Elfers C, Kratz M, Hoofnagle AN (2011). Vitamin d deficiency in obese children and its relationship to insulin resistance and adipokines. J Obes.

[36] Steinhoff JS, Lass A, Schupp M (2021). Biological functions of RBP4 and its relevance for Human diseases. Front Physiol.

[37] Metheniti D, Sakka S, Dracopoulou M, Margeli A, Papassotiriou I, Kanaka-Gantenbein C (2013). Decreased circulating 25-(OH) vitamin D concentrations in obese female children and adolescents: positive associations with Retinol binding Protein-4 and Neutrophil Gelatinase-associated Lipocalin. Horm (Athens).

[38] Rodrigues VS, Milanski M, Fagundes JJ, Torsoni AS, Ayrizono ML, Nunez CE (2012). Serum levels and mesenteric fat tissue expression of adiponectin and leptin in patients with Crohn’s disease. Clin Exp Immunol.

[39] Kahraman R, Calhan T, Sahin A, Ozdil K, Caliskan Z, Bireller ES (2017). Are adipocytokines inflammatory or metabolic mediators in patients with inflammatory bowel disease?. Ther Clin Risk Manag.

[40] Weigert J, Obermeier F, Neumeier M, Wanninger J, Filarsky M, Bauer S (2010). Circulating levels of chemerin and adiponectin are higher in ulcerative colitis and chemerin is elevated in Crohn’s disease. Inflamm Bowel Dis.

[41] Waluga M, Hartleb M, Boryczka G, Kukla M, Zwirska-Korczala K (2014). Serum adipokines in inflammatory bowel disease. World J Gastroenterol.

[42] Chouliaras G, Panayotou I, Margoni D, Mantzou E, Pervanidou P, Manios Y (2013). Circulating leptin and adiponectin and their relation to glucose metabolism in children with Crohn’s disease and ulcerative colitis. Pediatr Res.

[43] Ortega Moreno L, Sanz-Garcia A, de la Fernández MJ, Arroyo Solera R, Fernández-Tomé S, Marin AC (2020). Serum adipokines as non-invasive biomarkers in Crohn’s disease. Sci Rep.

[44] Walker GE, Ricotti R, Roccio M, Moia S, Bellone S, Prodam F (2014). Pediatric obesity and vitamin D deficiency: a proteomic approach identifies multimeric adiponectin as a key link between these conditions. PLoS ONE.

[45] Bidulescu A, Morris AA, Stoyanova N, Meng YX, Vaccarino V, Quyyumi AA (2014). Association between Vitamin D and Adiponectin and its relationship with body Mass Index: the META-Health study. Front Public Health.

[46] Vaidya A, Williams JS, Forman JP (2012). The independent association between 25-hydroxyvitamin D and adiponectin and its relation with BMI in two large cohorts: the NHS and the HPFS. Obes (Silver Spring).

[47] Husemoen LL, Skaaby T, Martinussen T, Jørgensen T, Thuesen BH, Kistorp C (2014). Investigating the causal effect of vitamin D on serum adiponectin using a mendelian randomization approach. Eur J Clin Nutr.

[48] Parikh S, Guo DH, Pollock NK, Petty K, Bhagatwala J, Gutin B (2012). Circulating 25-hydroxyvitamin D concentrations are correlated with cardiometabolic risk among American black and white adolescents living in a year-round sunny climate. Diabetes Care.

[49] Carvalho-Rassbach M, Alvarez-Leite JI, de Fátima (2019). Haueisen Sander Diniz M. is the association between vitamin D, adiponectin, and insulin resistance present in normal weight or obese? A pilot study. Clin Nutr Exp.

[50] Wright OR, Hickman IJ, Petchey WG, Sullivan CM, Ong C, Rose FJ (2013). The effect of 25-hydroxyvitamin D on insulin sensitivity in obesity: is it mediated via adiponectin?. Can J Physiol Pharmacol.

[51] Özkan B, Döneray H, Keskin H (2009). The effect of vitamin D treatment on serum adiponectin levels in children with vitamin D deficiency rickets. J Clin Res Pediatr Endocrinol.

[52] Nikooyeh B, Neyestani TR (2021). Can vitamin D be considered an adiponectin secretagogue? A systematic review and meta-analysis. J Steroid Biochem Mol Biol.

